# Convergent evolution in toothed whale cochleae

**DOI:** 10.1186/s12862-019-1525-x

**Published:** 2019-10-24

**Authors:** Travis Park, Bastien Mennecart, Loïc Costeur, Camille Grohé, Natalie Cooper

**Affiliations:** 1 Department of Life Sciences, Natural History Museum, Cromwell Road, SW7 5BD London, UK; 2 Naturhistorisches Museum Basel, Augustinergasse 2, 4001 Basel, Switzerland; 3 Naturhistorisches Museum Wien, Burgring 7, 1010 Vienna, Austria; 4 Division of Paleontology, American Museum of Natural History, Central Park West at 79th Street, New York, NY 10024 USA; 5 Laboratory Paleontology Evolution Paleoecosystems Paleoprimatology (PALEVOPRIM) – UMR 7262, CNRS-INEE/University of Poitiers, 86073 Poitiers Cedex 9, France

**Keywords:** Convergence, Odontoceti, Inner ear, Echolocation, Ecomorphology, Phylogenetic comparative methods

## Abstract

**Background:**

Odontocetes (toothed whales) are the most species-rich marine mammal lineage. The catalyst for their evolutionary success is echolocation - a form of biological sonar that uses high-frequency sound, produced in the forehead and ultimately detected by the cochlea. The ubiquity of echolocation in odontocetes across a wide range of physical and acoustic environments suggests that convergent evolution of cochlear shape is likely to have occurred. To test this, we used SURFACE; a method that fits Ornstein-Uhlenbeck (OU) models with stepwise AIC (Akaike Information Criterion) to identify convergent regimes on the odontocete phylogeny, and then tested whether convergence in these regimes was significantly greater than expected by chance.

**Results:**

We identified three convergent regimes: (1) True’s (*Mesoplodon mirus*) and Cuvier’s (*Ziphius cavirostris*) beaked whales; (2) sperm whales (*Physeter macrocephalus*) and all other beaked whales sampled; and (3) pygmy (*Kogia breviceps*) and dwarf (*Kogia sima*) sperm whales and Dall’s porpoise (*Phocoenoides dalli*). Interestingly the ‘river dolphins’, a group notorious for their convergent morphologies and riverine ecologies, do not have convergent cochlear shapes. The first two regimes were significantly convergent, with habitat type and dive type significantly correlated with membership of the sperm whale + beaked whale regime.

**Conclusions:**

The extreme acoustic environment of the deep ocean likely constrains cochlear shape, causing the cochlear morphology of sperm and beaked whales to converge. This study adds support for cochlear morphology being used to predict the ecology of extinct cetaceans.

## Background

Odontocetes (toothed whales) are the most successful lineage of marine mammal, with 75 extant species inhabiting every ocean, and several river systems [1]. The catalyst for their evolutionary success is echolocation - a complex form of biological sonar where high-frequency sounds are produced in the nasal passages and sent out into the surrounding environment [2]. The reflected signal is ultimately detected by the cochlea, allowing odontocetes to construct a mental model of their surroundings [3]. Previous studies have identified different echolocation types and cochlear morphologies, largely correlated with habitat, hearing abilities and phylogenetic relationships [4–7]. There is an emerging consensus that the shape of the cochlea is an excellent proxy to distinguish these in both extant and extinct taxa, allowing inferences to be made about ecology and phylogenetic position, even from fragmentary remains [7–9].

All extant toothed whales are believed to echolocate [5], and even the earliest known taxa are thought to have had the ability [8, 10–12], but they do so in a wide spectrum of environments; ranging from the complex, turbid and shallow waters occupied by river dolphins to the wide-open, clear, deep-water spaces that oceanic (pelagic) and deep diving species reside in. Different species also feed on different prey types, requiring varying degrees of resolution in their echolocation signal. Additionally, physical factors such as water temperature, depth and salinity all constrain sound speed through the aquatic medium and the wavelength of any given frequency [3]. We can therefore hypothesize that species occupying similar ecological niches will convergently evolve cochleae of a similar shape due to the auditory demands of that particular environment.

Here, we attempt to detect the presence of convergent evolution in a sensory organ using quantitative methods. We use cochlear shape data from a broad sample of odontocetes to test for convergent evolution of echolocatory capabilities in toothed whales. We first identify convergent regimes within the Odontoceti, then quantify the degree of convergence and its strength, and test for statistical significance using a variety of methods. Results are consistent with strong selective pressures related to living in an oceanic habitat and diving to extreme depths leading to the convergent evolution of particular cochlear shapes in different lineages. In contrast, the river dolphins, which display convergent evolution in other aspects of their morphology [13–17] do not appear to have convergently evolved a distinct cochlear shape. Habitat type and diving ability appear to be strong selection pressures on cochlear shape.

## Results

### Geometric morphometrics

**Fig. 1:**
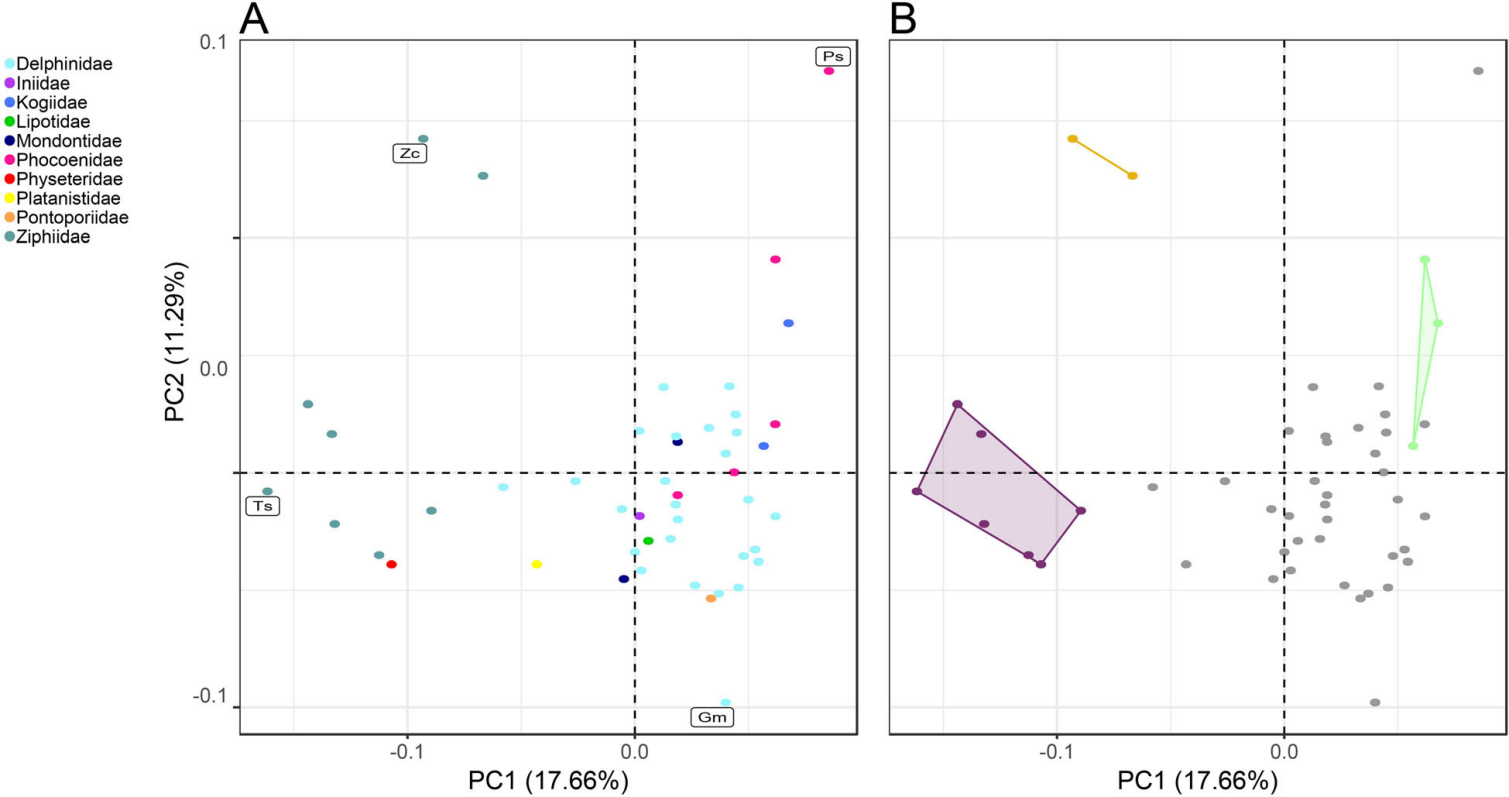
**a** Morphospace of cochlear shape in 48 toothed whale species, from principal component analysis (PCA) of 371 landmarks and semi-landmarks. Labelled points indicate cochleae of taxa at the four extremes of the morphospace: Ts: *Tasmacetus shepherdi*; Zc: *Ziphius cavirostris*; Gm: *Globicephala melas*; Ps: *Phocoena spinipinnis* (see Fig. 2). **b** The same morphospace with convergent groups mapped on. Colours correspond to convergent regimes: purple: (regime A) Ziphiid/Physeterid group; yellow: (regime B) *Ziphius*/*Mesoplodon mirus* group; green: (regime C) Kogiid/*Phocoenoides dalli* group; grey: non-convergent taxa. Percentages in brackets describe the amount of shape variation described by each principal component (PC)

Principal components analysis (PCA) of 371 landmarks describing cochlear shape variation in 48 species of odontocete revealed that beaked whales and dolphins have the widest range of cochlear shape variation in PC1 and beaked whales and porpoises have the widest range of shape variation in PC2 (Fig. 1).

**Fig. 2:**
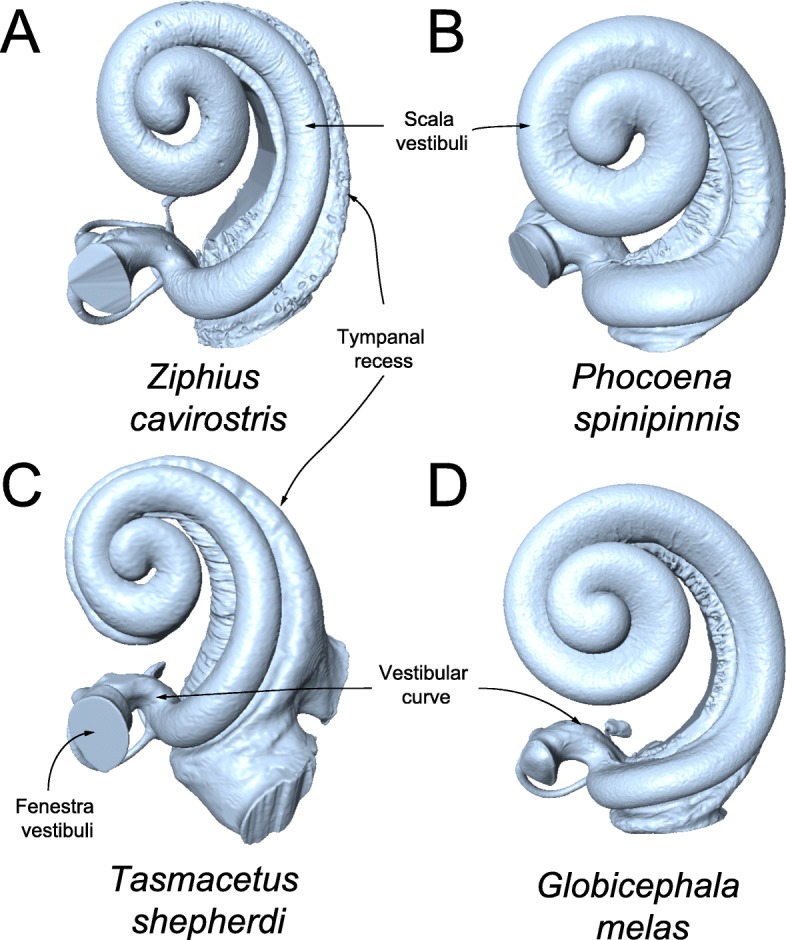
Cochleae of example taxa near the extremes of the morphospace shown in Fig. 1 (not to scale): **a** Zc: *Ziphius cavirostris*; **b** Ps: *Phocoena spinipinnis*; **c** Ts: *Tasmacetus shepherdi*; **d** Gm: *Globicpehala melas*

Principal component 1 (PC1) accounted for 17.66% of the cochlear shape variation (Fig. 1). The negative values of this axis represents cochleae with: 1) a radially expanded scala tympani (i.e. a tympanal recess); 2) a scala vestibuli that is transversely (radially) thinner along its length; 3) a larger fenestra vestibuli; and 4) a vestibular curve (sensu Luo & Marsh [18]) that does not extend as far dorsally. The positive values of this axis represents cochleae with: 1) a scala tympani that is not radially inflated; 2) a relatively thicker scala vestibuli; 3) a smaller fenestra vestibuli; and 4) a more dorsally extended vestibular curve. PC2 accounted for 11.29% of the cochlear shape variation. The negative values of this axis represent cochleae with an oval-shaped fenestra vestibuli with the long axis oriented approximately anteropostosteriorly. The positive values of this axis represents cochleae with a circular fenestra vestibuli (Figs. 1 and 2).

### Phylogenetic signal

We found a statistically significant phylogenetic signal in the PCs for cochlear shape (K_mult_ = 0.3009, *p*-value = 0.001).

### Identifying convergent regimes

**Fig. 3:**
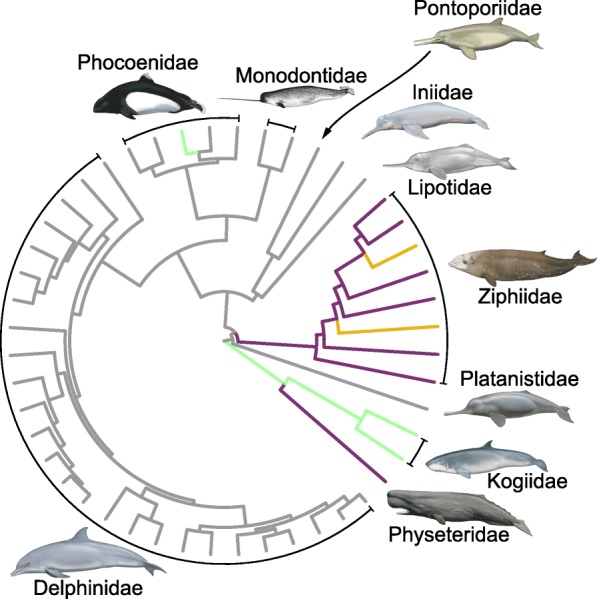
Results of SURFACE analysis of the cochlear morphology of 48 toothed whale species. This shows the phylogenetic tree of Steeman et al. [19], pruned to taxa sampled in this study only, with convergent regimes painted on branches. Convergent regimes are as follows: purple: (regime A) Physeteridae and Ziphiidae (except *Mesoplodon mirus* and *Ziphius cavirostris*); yellow: (regime B) *Mesoplodon mirus* and *Ziphius cavirostris*; and green: (regime C) Kogiidae and *Phocoenoides dalli*. Grey branches show species that are not members of any convergent regime. Illustrations showing representative members of each odontocete family drawn by Carl Buell, used with permission

The SURFACE analysis identified a total of five distinct evolutionary regimes in the cochleae shape data, three of which were convergent (Figs. 1 and 3; Table 1). These regimes were: 1) sperm whale (*Physeter macrocephalus*) and all other beaked whales (ziphiids; regime A; Figs. 1 and 3); 2) True’s beaked whale (*Mesoplodon mirus)* and Cuvier’s beaked whale (*Ziphius cavirostris*; regime B; Figs. 1 and 3); and 3) kogiids, dwarf sperm whale (*Kogia sima*) and pygmy sperm whale (*Kogia breviceps*), and Dall’s porpoise (*Phocoenoides dalli*; regime C; Figs. 1 and 3). Despite generally being assumed to be convergent, the ‘river dolphins’ did not form a convergent regime.

**Table 1:** Results of SURFACE analysis of 48 toothed whale species cochlear shapes

	Multipeak OU		OU1		BM	
Model outputs	Value		Value		Value	
AICc	− 366		− 313		− 174	
Phenotypic regimes	5		1		–	
Phenotypic regime shifts	8		1		–	
Convergent phenotypic regimes	3		–		–	
Convergent phenotypic regime shifts	6		–		–	
Convergence fraction	0.75		–		–	
Parameters	PC1	PC2	PC1	PC2	PC1	PC2
α	0.433	3.055	0.013	0.161	–	–
t_1/2_	1.560	0.227	53.915	4.294	–	–
σ^2^	0.001	0.005	0.000	0.001	0.000	0.000
θ_a_	0.064	0.055	−0.032	0.003	–	–
θ_b_	0.098	0.171	–	–	–	–
θ_c_	−0.126	− 0.011	–	–	–	–
θ_e_	−0.079	0.134	–	–	–	–
θ_h_	0.021	−0.015	–	–	–	–

Models were fitted to the cochlear shape described by principal component (PC) 1 and PC2. Multipeak Ornstein Uhlenbeck (OU): convergent OU model fitted by backward phase of SURFACE; OU1: single peak OU model; BM: Brownian motion model

We found the best model in SURFACE for explaining cochlear evolution was the multipeak Ornstein-Uhlenbeck (OU) model, which had an AICc score of − 365.57. The Brownian motion (BM) and single peak OU models had AICc scores of − 173.88 and − 312.98 respectively (Table 1). PC2 had much larger and smaller values for the rate of adaptation to optima (α) and the expected time to evolve halfway to an optimum (t_1/2_), respectively.

### Detecting significant levels of convergence

We used Stayton’s C metrics (convergence metrics; see Methods) [20] to statistically test for convergence. In regimes A and B all C-metrics were significant, (Table 2) with C_1_ values indicating that an average phenotypic distance of 35 and 21.6% has been closed by convergence for regimes A and B, respectively. Conversely, there were no statistically significant C-metrics in the regime C, with an average phenotypic distance of 21.1% closed by convergence. We did not find any statistically significant C-metrics in river dolphin cochleae.

**Table 2:** C_1_ - C_4_ convergence measures and *p*-values for each convergent regime

Regime	Variable	C1	C2	C3	C4
A	C-value	0.216	0.053	0.103	0.006
	*p*-value	**0**	**0**	**0**	**0**
B	C-value	0.350	0.116	0.201	0.132
	*p*-value	**0.003**	**0**	**0**	**0**
C	C-value	−0.211	−0.034	− 0.071	− 0.004
	*p*-value	0.999	0.999	0.999	0.999

*P*-values were derived from 1000 simulations to test the hypothesis that the observed values are greater than random simulations based on Brownian motion. Significant *p*-values are in bold

### Investigating the strength of convergence

None of the convergent regimes found in the SURFACE analysis had statistically significant Wheatsheaf index values (Table 3). Note this means only that the convergence is not unusually strong in these regimes, not that they are not convergent.

**Table 3:** Results of the Wheatsheaf index analysis for each convergent regime

Regime	WI value	*p*-value	95% CI
A	1.112	0.955	1.054–1.256
B	1.432	0.46	1.390 - ∞
C	1.632	0.335	1.584–3.470

*WI* Wheatsheaf index, *CI* 95% confidence intervals on WI. Note that the upper CI for regime B is infinity, because only two taxa are being used to perform the calculation resulting in a division by zero

### Investigating potential mechanisms for convergence

**Fig. 4:**
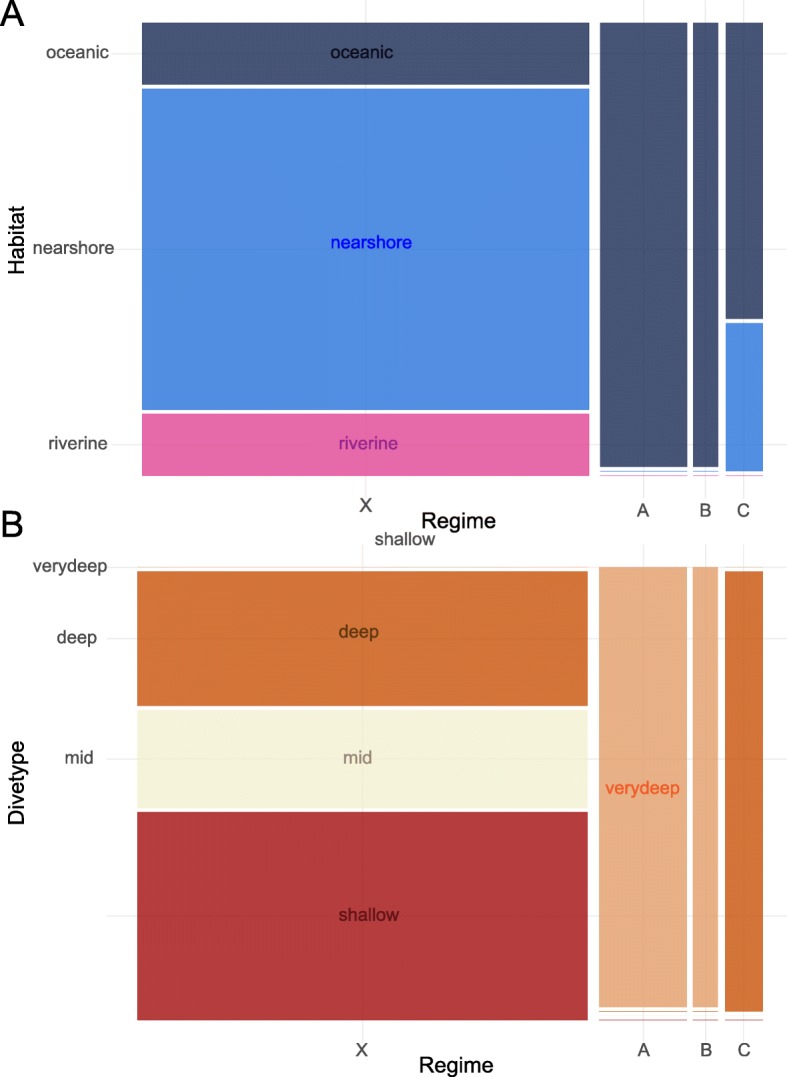
Mosaic plots of: **a** Habitat type and regime membership (i.e. regime A: purple, regime B: yellow or regime C: green regime. Regime X contains taxa that do not belong to any of the three convergent regimes); and **b** Dive type and regime membership; showing how the convergent regimes are dominated by taxa living in oceanic habitats and diving to extreme depths, respectively. The width of each regime type is proportional to the number of taxa it contains. The height of each colour is proportional to the number of taxa in that ecological subcategory. Lines indicate that no species belong in that category. Colours represent ecological subcategories: riverine: pink; nearshore: blue; oceanic: navy; shallow: red; mid: ivory; deep: dark orange; very deep: light orange

There was a significant association between being a member of regime A and living in an oceanic habitat and diving to extreme depths (> 1000 m i.e. ‘very deep’ dive type; Fig. 4). However, no other ecological associations were significant after Bonferroni correction. Although species in the convergent regimes were overall significantly larger in body size than those not within a convergent regime (ANOVA: F_3,44_ = 4.033, *p*-value = 0.013), the species in the three convergent regimes did not have significantly different body sizes (ANOVA: F_2,9_ = 3.239, *p*-value = 0.087).

## Discussion

In this study, we investigated convergent evolution in the cochlear shape of toothed whales. The aim was to detect convergent evolution in cochlear shape without any a priori information on which taxa might be convergent. Using a SURFACE analysis (bearing in mind the assumptions of independence in each trait; see Methods), we found three convergent phenotypic regimes that have evolved independently in different lineages, two of which were significantly convergent, i.e. more similar to each other than to their ancestors [20]. The first significant convergent regime (regime A) consists of the sperm whale and all the beaked whales sampled in this study, excluding True’s beaked whale (*Mesoplodon mirus*) and Cuvier’s beaked whale (*Ziphius cavirostris*). Regime A taxa share very similar ecologies. They inhabit oceanic regions, rarely venturing close to the shore. They are deep diving animals, capable of staying submerged for over an hour [21–23]. They are also suction feeders; teeth, if present are non-functional, unnecessary or are used for intraspecific interactions such as sexual displays (with the exception of *Tasmacetus*) [24–26]. The cochleae of the sperm whale and extant beaked whales also have a tympanal recess, a feature not seen in other living odontocetes. The function of this feature, which is a radial expansion of the scala tympani, is still unknown (but see Park et al. [27] for possible explanations), but it is also present in baleen whales (except for balaenids) [28], confounding functional explanations related to the ecology of this convergent regime [29].

Functionally, the auditory pathway of sperm whales and beaked whales (as well as kogiids) are different to that of other odontocetes. They possess a singular middle ear type [30] where mallear morphology is distinct, the middle ear bones are relatively rigid and the tympanoperiotic complex is synotosed, all preventing bending and rotation of the earbones. Additionally, sperm whales and beaked whales also retain a bony connection of their earbones to the skull through the posterior process, unlike the ligamentous suspension system seen in other odontocetes [31], hinting at the existence of a bone conduction mechanism in these taxa.

The remaining two beaked whale taxa from this study, True’s and Cuvier’s beaked whales, form the second significantly convergent regime (regime B). It is unclear what separates these two species from those of the previous regime. They are thought to have the same deep-diving, suction feeding ecology as all other beaked whales; indeed, Cuvier’s beaked whale has the greatest known dive depth of any cetacean (or mammal) of 2992 m [32]. Both taxa also have a tympanal recess and share the derived auditory pathway morphology described above. True’s beaked whale is one of the most poorly known cetaceans, making comparisons of the ecology in this regime difficult.

The dwarf and pygmy sperm whales (*Kogia* spp.) and Dall’s porpoise (*Phocoenoides dalli*)*,* were unexpectedly recovered as a third convergent regime (regime C), although the convergence was not statistically significant. Nevertheless, an examination of their ecologies does reveal similarities. Kogiids are rarely sighted at sea, due to their preference for oceanic habitats [33–35]. Porpoises, conversely, generally prefer shallow waters over the continental shelf, with some species even living in rivers. Interestingly, *P. dalli* is one of only two exceptions to this trend, inhabiting deep oceanic waters, only coming close to shore when there are deep-water trenches present [36]. Additionally, Dall’s porpoise is the largest member of the porpoise family, reaching lengths of up to 2.4 m, similar in size to at least the dwarf sperm whale (the pygmy sperm whale is larger, with a minimum size of 2.7 m) [36]. All species in this regime are suction feeders; the pygmy sperm whale has been experimentally observed employing rapid gape and gular kinematics to generate strong suction [37, 38]. Dall’s porpoise has tiny, non-functional teeth, and therefore instead relies on suction to acquire prey, similar to other porpoises [14, 39]. Interestingly, kogiids use narrow band high frequency echolocation signals, the same as porpoises, differing from their close relatives, sperm whales that use multi-pulsed signals [40]. Both pygmy and dwarf sperm whales are thought to be capable of very deep dives, with observed dives of over 50 min duration recorded previously [41]. Maximum dive depth is unknown, but some prey items are only known to inhabit depths of 500–1300 m [42, 43]. Similarly, the maximum dive depth of Dall’s porpoise is unknown (Hanson & Baird [44] observed a dive of 94 m), but the abundance of deep-water prey in its diet and physiological factors such as a high blood oxygen content suggest that it also dives to great depths [45]. The convergence of cochlear shape with pygmy and dwarf sperm whales also adds evidence to the hypothesis that Dall’s porpoise is capable of diving considerably deeper than has been previously recorded. However, the two genera differ in the morphology of their auditory pathway, with pygmy and dwarf sperm whales sharing the same morphology as beaked whales and sperm whales whereas Dall’s porpoise possesses the ligamentous system seen in all other odontocetes; only the morphology of the cochlea appears to be convergent.

The two statistically significant regimes found (A and B) above suggest there is a strong selection pressure on taxa that have an oceanic ecology characterized by diving to extreme depths. This is further corroborated by the significant associations of living in oceanic habitats, diving to extreme depths and belonging to the regime A. Despite all taxa in this regime also being specialist suction feeders, suction feeding occurs across odontocetes so is therefore not associated with any single convergent regime. Larger odontocetes have a larger maximum prey detection range and inspection range [40], which is likely to be very important for sperm whales and beaked whales, who traverse large distances in search of patchy prey [46, 47]. The morphology of these convergent regimes also appears to have converged rapidly, as shown by the respective large and small values for rate of adaptation to an optimum (α) and the expected time to evolve halfway to an optimum (t_1/2_) of PC2 (representing fenestra vestibuli shape) in the SURFACE analysis (Table 1). This indicates that the fenestra vestibuli converged on the same morphology more rapidly than other aspects of cochlear morphology. Sound initially reaches the cochlea through this opening, potentially causing the morphology to change more rapidly than elsewhere. Additional selection pressures could potentially come from the physical properties of the water itself. The speed of sound in water depends on the temperature, salinity, depth and time from source. Sound velocity decreases with temperature until it reaches its minimum around 1000 m depth. At this point sound velocity begins to increase with depth again due to the increasing pressure, eventually becoming faster than surface speeds around 2500 m depth [48], depths that only sperm whales and beaked whales are capable of reaching. The high pressure may also have an influence on the hydrodynamics of the fluids of the inner ear, resulting in the cochlear morphologies seen, although well-developed vascular structures in the pterygoid sinus are thought to maintain pressures surrounding the ear region [49, 50]. It is therefore possible that the convergence seen in cochlear morphology between sperm whales and beaked whales is an adaptation for dealing with the particular acoustic environment found at these depths, although it is currently unclear how the mechanism would work. It is also likely that the extreme environment at these depths has driven convergent evolution in other marine lineages e.g. the independent evolution of bioluminescence in deep-sea fish, cnidarians and cephalopods [51]. It is also entirely possible that there is no underlying functional or ecological mechanism behind these similarities; not all traits are adaptive [52].

Previous analyses of convergent evolution in odontocetes have focused on the morphological similarity of the distantly related ‘river dolphin’ genera, *Platanista, Lipotes, Inia* and *Pontoporia* [13–17, 53]. Interestingly, our results detected no significant convergence in river dolphin cochleae, despite previous results showing that the cochleae of freshwater cetaceans separate out from marine taxa in a canonical variate analysis [7]. It is possible that there is convergence among a subsample of the ‘river dolphin’ genera, but this was not tested for in this study. Furthermore, our increased sample size includes additional taxa that spend some or all of their time in rivers, which may prevent a convergent signal being detected.

More generally, it is clear from our study that cochlear shape is not dominated by a single or even several, major axes of variation (PC1 accounts for only 17.66%). Variation is widely spread among many PCs. Caution should therefore be used when interpreting relationships between PC scores and changes in cochlear shape. Costeur et al. [7] also found similar patterns when using highly dimensional data. This contrasts with studies on skull shape, where length and width are often the primary sources of variation (e.g. [53]).

Potential caveats of this study are the assumptions of independent rates of adaptation and diffusion in the OU models used in the SURFACE analysis [54], with the resulting use of only the first two PCs for the analyses. It is likely that analyses using more PCs, or indeed all shape variables, would reveal different patterns. However, at present there is no suitable method for this kind of highly dimensional data. Additionally, we may have found different results if we had manually selected groups of taxa to test for convergence a priori, rather than using the approach we used here*.* Another issue was that obtaining ecological data on these reclusive animals, in particular for the deepest diving odontocetes, is incredibly difficult. Therefore, our ecological characterizations are necessarily broad. More accurate information on variables such as dive depth may help to narrow down the selective pressures driving inner ear evolution. In future, the inclusion of fossil taxa may also identify convergent evolution over even longer timescales than that found here.

## Conclusions

Previously, morphological convergence in vertebrates has been investigated using observational evidence e.g. [55], skull shape and linear morphometric measurements e.g. Mahler et al. [56]; Esquerre & Keogh [57]; Page & Cooper [17]; Morris et al. [58]; or qualitative comparisons e.g. [59]. Whilst the cochlea has been shown to demonstrate convergence at the genetic level in prior studies on echolocating mammals [60–63], here we demonstrate the usefulness of the cochlea as a means of quantitatively testing for morphological convergence. This novel use of the cochlea, enabled by the increased ease of access to high resolution X-ray tomography facilities, adds to its great value in comparative studies, having previously been used as an indicator of hearing ability [5, 8], phylogenetic position [64] and habitat preference [7, 65]. The extraordinary demands of accessing, navigating, communicating and searching for prey in the deep ocean strongly constrains the possible range of phenotypes that can be expressed. We hypothesise that this extreme acoustic environment selects for a particular cochlear shape, one that we demonstrate has evolved convergently in disparate odontocete lineages. Future studies should aim to incorporate fossil taxa into their samples with the possibility of using these methods (in addition to seeking methodological improvements in dealing with highly dimensional data) to reconstruct their ecology and investigate the timing of convergent evolution in echolocatory capabilities driven by geographic and oceanic changes during the past 30 million years.

## Methods

### Data collection

We obtained microCT scans of the periotics - the bone containing the inner ear - of 48 species (comprising 94% of extant genera) of odontocetes by imaging osteological specimens from museum collections (see Additional file 1). Using this data, we reconstructed 3D models of the inner ears using the segmentation and thresholding editors in Avizo 9.0 [66], and then cleaned the resulting 3D models using Geomagic Wrap^Ⓡ^ [67]. Next we landmarked the digital models with 40 sliding semilandmark curves comprising a total of 371 landmarks (see Additional file 1), using IDAV Landmark [68]. The position of these curves followed the protocols of Costeur et al. [7], using only the curves from the cochlea and the vestibular aqueduct because 1) the focus of this study is hearing ability, i.e. echolocation rather than balance; and 2) the semi-circular canals are not phylogenetically or ecologically informative in odontocetes [7]. Landmarks were placed by a single investigator (TP) to avoid multi-user bias in placement. Finally we exported coordinates from the landmarked models as .pts files from IDAV Landmark [68]. Terms of cochlear orientation refer to the spiral itself rather than in relation to the body of the animal.

For phylogenetic analyses we used the time-calibrated tree of Steeman et al. [19], a robust molecular phylogeny derived from mitochondrial and nuclear markers, pruned to the 48 taxa sampled in this study.

### Geometric morphometrics

We performed all geometric morphometric analyses in R version 3.4.3 [69], using the R package GEOMORPH [70]. First, we used Generalised Procrustes Analysis (GPA) to remove the effects of position, scale and orientation. The semilandmarks were ‘slid’ along their tangent vectors between adjacent semilandmarks until their positions minimised the shape difference between specimens (using the Procrustes distance criterion), to reduce the effect of their initially arbitrary placement [71–73]. We then performed a principal component analysis (PCA) on the resulting Procrustes coordinates using the ‘plotTangentSpace’ function; and use these principal components (PCs) in all further analyses.

### Phylogenetic signal

To determine whether close relatives tend to have more similarly shaped cochlea than more distant relatives, we estimated phylogenetic signal in our PC scores using the *K*_mult_ statistic. This method is designed to deal with high-dimensional multivariate data (e.g. landmark configurations) by exploiting the statistical equivalency between covariance-based and distance-based approaches for Euclidean data [74]. We calculated this statistic using all PC scores.

### Identifying convergent regimes

In this study, we aimed to identify odontocete cochleae that have convergently evolved a similar shape without defining groups as convergent a priori. To identify convergent regimes in odontocete cochleae we used the R package SURFACE [75]. The SURFACE approach uses an Ornstein-Uhlenbeck (OU) process – a random walk where trait values are pulled back towards an adaptive peak/long term mean – to identify groups that share a similar adaptive peak, and can hence be defined as belonging to the same convergent regime Mahler et al. [56]. A SURFACE analysis is split into a forward phase and a backward phase to firstly locate regime shifts on a tree, and secondly identify whether the shifts are convergent. In the forward phase, SURFACE starts with the simplest model where the entire clade is in a single adaptive regime. SURFACE then adds regime shifts one at a time to the origin of each branch, and the branch with the lowest small sample size corrected Akaike Information Criterion (AICc) score is retained. Regime shifts continue to be added until there is no change in AICc (i.e. change in AICc is 0). In the backward phase, the final model from the forward phase is simplified by pairwise collapses of regimes into one convergent regime. If this improves the AICc score, the model simplification continues using this model. Regime shifts continue to be collapsed until there is no change in AICc. This model represents the final set of convergent regimes. We then compared the fit of this model to a Brownian motion (BM) model and a OU model with a single peak (OU1) to ensure that these simpler models did not fit better with our shape data (i.e. PC scores).

We note that Zelditch et al. [54] caution against using SURFACE with high-dimensional data because it assumes that each trait has an independent rate of adaptation (α) and diffusion (σ^2^). Ingram and Mahler [75] also suggest that between two and four traits should be used, as large numbers of traits may be difficult to interpret biologically and are unlikely to be involved in biological adaptation. To minimise these effects, here we use the first two principal components (see Additional file 1 for results of analyses using 3 PCs and 4 PCs) as the traits for our SURFACE analysis. Note that, we use our SURFACE analyses to identify putatively convergent groups (or regimes) only; we do not use SURFACE alone to define these groups as convergent.

### Detecting significant levels of convergence

Using the convergent regimes identified using SURFACE, we next used the measures proposed by Stayton [20] in the R package CONVEVOL to quantify the degree of convergence in each putatively convergent group and test for statistical significance. We also additionally tested for convergence in ‘river dolphin’ (*Platanista, Lipotes, Inia* and *Pontoporia*) cochleae as previous analyses had found morphological convergence in skull shape and association of cochlear shape with habitat preference [7, 17]. The C-metrics (C_1_ - C_4_), are distance-based measures that define convergence as where *two or more taxa have evolved to be more similar to one another than their ancestors were to each other* [20, 76]. C_1_ represents the proportion of the maximum phenotypic distance between two convergent lineages that has been reduced by subsequent evolution (i.e. relative amount of convergence), ranging from 0 to 1 and increasing as the degree of convergence increases. The maximum phenotypic distance is estimated using ancestral state estimation under a BM model. C_2_ is similar to C_1_, but is not scaled so the magnitude of the evolutionary change can be taken into account (i.e. absolute amount of convergence). C_3_ and C_4_ standardise C_2_ by dividing it by the total amount of evolution that has taken place in the clade containing the convergent taxa and the whole phylogeny respectively. We used the first 30 PCs to calculate these values as this represents 95% of the total variation in cochlea shape. Significance for these metrics is calculated by simulating evolutionary changes 1000 times via BM using the phylogeny and a variance-covariance matrix derived from the observed data (i.e. the PC scores) as the rate of evolution. C_1_ - C_4_ are calculated for each simulated dataset, creating an expected distribution of each metric (the higher the metric the stronger the convergence); the *p*-value is the proportion of times the simulated value exceeds the observed value.

### Investigating the strength of convergence

Stayton’s [20] C-metrics measure the significance of convergence, but do not measure strength of convergent evolution, therefore we calculate this using the Wheatsheaf index in the WINDEX R package [77], using the convergent regimes identified in the SURFACE analysis as the focal groups. The Wheatsheaf index is calculated by dividing the mean corrected phenotypic distance matrix for pairwise comparisons between all species in the phylogeny, by the mean corrected phenotypic distance for pairwise comparisons between focal species only. As with the C-metric analyses, the first 30 PCs were used. Additionally, 95% confidence intervals (CI) were obtained by jack-knifing the data and calculating the intervals from the resulting distribution. Confidence intervals are given because the calculation of the Wheatsheaf Index is not amenable to multiple, independent sampling (it uses information from the entire sample). We also obtained a *p*-value for the Wheatsheaf index values by bootstrapping the trait values (i.e. PC scores) at the tips of the phylogeny and recalculating the Wheatsheaf index to give a distribution of values. The proportion of these bootstrapped values that are greater than or equal to the observed Wheatsheaf index is the *p*-value.

### Investigating potential mechanisms for convergence

Finally, once convergence regimes were identified and quantified, we also wanted to determine whether particular ecological factors were correlated with membership of these regimes to highlight potential drivers underlying the convergence. Significant correlations may be indicative of selection pressures related to these ecological factors driving convergence in cochlear morphology. To do this we collated ecological data on habitat (riverine, nearshore, oceanic), diet (generalist, fish, cephalopods), feeding behaviour (raptorial, suction) and dive type (shallow (estimated max dive depth ≤ 100 m), mid (estimated max dive depth ~ 500 m), deep (estimated max dive depth ~ 1000 m), very deep (estimated max dive depth > 1000 m)) for each species using Mittermeier & Wilson [78], Jefferson et al. [79], McCurry et al. [53], Hocking et al. [26, 80] and multiple additional sources (see Additional file 1).

We tested for an association among ecological categories and each convergent regime using *χ*^2^ tests. To deal with species that belonged to multiple categories, for example nearshore and oceanic, we repeated the analyses with the species first analysed as nearshore then as oceanic. This led to six *χ*^2^ tests for each regime, so we corrected the resulting *p-*values using a Bonferroni correction to reduce the likelihood Type I error due to multiple testing. We also investigated whether the regimes contained species with significantly different body sizes using analysis of variance (ANOVA) of natural log transformed body masses.

## Supplementary information

**Additional file 1: Table S1.** Specimens used in this study. Institutional abbreviations: AMNH, American Museum of Natural History, New York, USA; IRNSBV, Belgian Royal Institute of Natural Sciences, Brussels, Belgium; NMB, Naturhistorisches Museum Basel, Basel, Switzerland; NHMUK, Natural History Museum, London, England; NMVC, Museums Victoria, Melbourne, Australia. **Figure S1.** Cochlea of *Cephalorhynchus commersonii* (NHMUK1952.6.20.4.2) in: (A) vestibular; (B) anterior; (C) dorsal; and (D) lateral views, showing placement of landmarks for this study. **Table S2.** Results of *χ*^2^ analysis. *Χ*^2^: chi-squared value; df: degrees of freedom; bc: Bonferroni corrected *p*-value. **Table S3.** Results of the SURFACE analysis using 3 PCs. Parameters were found by the evolutionary models fitted to the evolution of cochlear shape in toothed whales described by PC1, PC2 and PC3. **Table S4.** Results of the SURFACE analysis using 4 PCs. Parameters were found by the evolutionary models fitted to the evolution of cochlear shape in toothed whales described by PC1, PC2, PC3 and PC4. **Table S5.** C_1_ - C_4_ convergence measures and *p*-values using 3 PCs. *P*-values were derived from 1000 simulations to test the hypothesis that the observed values are greater than random simulations based on Brownian motion. Significant values in bold. **Table S6.** C_1_ - C_4_ convergence measures and *p*-values using 4 PCs. *P*-values were derived from 1000 simulations to test the hypothesis that the observed values are greater than random simulations based on Brownian motion. Significant values in bold. **Table S7.** Results of the Wheatsheaf index analysis using 3 PCs. WI: Wheatsheaf index. **Table S8.** Results of the Wheatsheaf index analysis using 4 PCs. WI: Wheatsheaf index. **Figure S2.** Cladogram showing the phylogenetic relationships of the taxa in this study.

## Data Availability

All data are available on the NHM Data Portal (Park et al. 2018; 10.5519/0082968), and all code needed to reproduce these analyses is available on GitHub at https://github.com/travispark/Odontocete-inner-ear-convergence. [We will add a Zenodo DOI when the paper is accepted].

## Abbreviations

AIC: Akaike Information Criterion
*ANOVA*: Analysis of variance
BM: Brownian motion
C-metric: Convergence metric
GPA: Generalised Procrustes analysis
OU: Ornstein-Uhlenbeck
PC: Principal component
PCA: Principal component analysis
t_1/2_: Expected time to evolve halfway to an optimum
α: Rate of adaptation
σ^2^: Rate of diffusion
