# Supplementary material for: Convergent evolution in toothed whale cochleae
Source: BMC Evol Biol. 2019 Oct 24;19:195. doi: 10.1186/s12862-019-1525-x (PMC6813997; doi:10.1186/s12862-019-1525-x)
Supplement: Supplementary file 1 — Additional file 1: Table S1. Specimens used in this study. Institutional abbreviations: AMNH, American Museum of Natural History, New York, USA; IRNSBV, Belgian Royal Institute of Natural Sciences, Brussels, Belgium; NMB, Naturhistorisches Museum Basel, Basel, Switzerland; NHMUK, Natural History Museum, London, England; NMVC, Museums Victoria, Melbourne, Australia. Figure S1. Cochlea of Cephalorhynchus commersonii (NHMUK1952.6.20.4.2) in: (A) vestibular; (B) anterior; (C) dorsal; and (D) lateral views, showing placement of landmarks for this study. Table S2. Results of χ2 analysis. Χ2: chi-squared value; df: degrees of freedom; bc: Bonferroni corrected p-value. Table S3. Results of the SURFACE analysis using 3 PCs. Parameters were found by the evolutionary models fitted to the evolution of cochlear shape in toothed whales described by PC1, PC2 and PC3. Table S4. Results of the SURFACE analysis using 4 PCs. Parameters were found by the evolutionary models fitted to the evolution of cochlear shape in toothed whales described by PC1, PC2, PC3 and PC4. Table S5. C1 - C4 convergence measures and p-values using 3 PCs. P-values were derived from 1000 simulations to test the hypothesis that the observed values are greater than random simulations based on Brownian motion. Significant values in bold. Table S6. C1 - C4 convergence measures and p-values using 4 PCs. P-values were derived from 1000 simulations to test the hypothesis that the observed values are greater than random simulations based on Brownian motion. Significant values in bold. Table S7. Results of the Wheatsheaf index analysis using 3 PCs. WI: Wheatsheaf index. Table S8. Results of the Wheatsheaf index analysis using 4 PCs. WI: Wheatsheaf index. Figure S2. Cladogram showing the phylogenetic relationships of the taxa in this study. [file 12862_2019_1525_MOESM1_ESM.docx]

**Electronic Supplementary Material for: Convergent evolution in toothed whale cochleae**

**Specimen List**

Table S1. Specimens used in this study. Institutional abbreviations: AMNH, American Museum of Natural History, New York, USA; IRNSBV, Belgian Royal Institute of Natural Sciences, Brussels, Belgium; NMB, Naturhistorisches Museum Basel, Basel, Switzerland; NHMUK, Natural History Museum, London, England; NMVC, Museums Victoria, Melbourne, Australia.

| Taxon | Specimen number | Resolution (μm) |
| --- | --- | --- |
| *Cephalorhynchus commersonii* | NHMUK1952.6.20.4 | 32.08 |
| *Cephalorhynchus heavisidii* | NHMUK1948.7.27.1 | 32.08 |
| *Delphinus delphis* | NMB6679 | 40 |
| *Feresa attenuata* | NHMUK1874.11.25.1 | 47.52 |
| *Globicephala melas* | NHMUK1947.12.31.4 | 32.11 |
| *Grampus griseus* | NHMUK1920.12.16.1 | 25.72 |
| *Lagenodelphis hosei* | NHMUK1895.5.9.1 | 38.75 |
| *Lagenorhynchus acutus* | NHMUK1928.19 | 32.27 |
| *Lagenorhynchus albirostris* | NHMUK1848.7.12.12 | 32.08 |
| *Lagenorhynchus australis* | NHMUK1944.11.30.1 | 32.27 |
| *Lagenorhynchus cruciger* | NHMUK1960.8.24.1 | 32.27 |
| *Lagenorhynchus obliquidens* | NHMUK1966.10.25.1 | 39.75 |
| *Lagenorhynchus obscurus* | NHMUK1841.1733 | 39.75 |
| *Orcaella brevirostris* | NHMUK1883.11.20.2 | 32.11 |
| *Orcinus orca* | NHMUK1927.28 | 39.84 |
| *Peponocephala electra* | NHMUK1992.100 | 32.08 |
| *Pseudorca crassidens* | NHMUK1992.248 | 39.84 |
| *Sotalia fluviatilis* | NHMUK1856.8.2.2 | 43.75 |
| *Sotalia guianensis* | IRSNBV20137 | 30.47 |
| *Sousa chinensis* | NHMUK1914.1.14.1 | 32.27 |
| *Stenella attenuata* | NHMUK1990.98 | 37.66 |
| *Stenella coeruleoalba* | NHMUK1940.3.2.1 | 37.66 |
| *Stenella longirostris* | NHMUK1990.104 | 32.27 |
| *Steno bredanensis* | NMVC36961 | 32.97 |
| *Tursiops aduncus* | NHMUK1882.1.2.3 | 39.75 |
| *Tursiops truncatus* | NHMUK1866.8.7.1 | 32.27 |
| *Inia geoffrensis* | NMB7167 | 30 |
| *Kogia breviceps* | NMVC24976 | 33.07 |
| *Kogia sima* | NHMUK1952.8.28.1 | 32.27 |
| *Lipotes vexillifer* | AMNH57333 | 30.7 |
| *Delphinapterus leucas* | NMBCIII1086 | 40 |
| *Monodon monoceros* | AMNH73315 | 40.69 |
| *Neophocaena phocaenoides* | NHMUK1903.9.12.3 | 32.27 |
| *Phocoena dioptrica* | NHMUK1939.9.30.1 | 38.75 |
| *Phocoena phocoena* | NMVC27654 | 33.08 |
| *Phocoena spinipinnis* | IRSNBV21219 | 27.35 |
| *Phocoenoides dalli* | NHMUK1965.1.19.2 | 43.75 |
| *Physeter macrocephalus* | NHMUK893 | 42.14 |
| *Platanista gangetica* | NMVC27417.2 | 41.5 |
| *Pontoporia blainvillei* | MNHN1934.375 | 38 |
| *Berardius arnuxii* | NHMUK1982.315 | 49.67 |
| *Hyperoodon ampullatus* | NHMUK1862.12.2.2 | 41.06 |
| *Mesoplodon bidens* | IRSNB16232 | 35 |
| *Mesoplodon grayi* | NMVC31378 | 44.02 |
| *Mesoplodon hectori* | NHMUK1876.2.16.3 | 30.11 |
| *Mesoplodon mirus* | NHMUK1920.5.20.1 | 47.52 |
| *Tasmacetus shepherdi* | NMVC37967.6 | 58.28 |
| *Ziphius cavirostris* | NHMUK1915.7.20.1 | 33.57 |

**Landmark/curve Definitions**

*Fenestra vestibuli*

Curves 0 – 3: 4 curves (1 per quarter): starts at midline of vestibular curve, travels anticlockwise (in vestibular view), at level of the point where the lateral semi-circular canal meets the fenestra vestibuli, finishes on same point

*Cochlear canal*

Curves 4 – 11: 8 curves on medial-most outline of scala vestibuli: starts where vestibular curve meets cochlear canal, finishes on apex

Curves 12 – 19: 8 curves on ventral-most outline of scala vestibuli: starts where vestibular curve meets cochlear canal, finishes on apex

Curves 20 – 30: 11 curves lateral most outline of scala vestibuli (starting at dorsal-most point of scala vestibuli), finishes on apex

Curves 31 – 39: 9 curves dorsal-most outline of scala tympani (starting just anterior to where cochlear aqueduct joins cochlear canal), finishes on apex

*Vestibular aqueduct*

Curve 40: 1 curve along its length ending at endolymphatic sac: runs along anterior surface


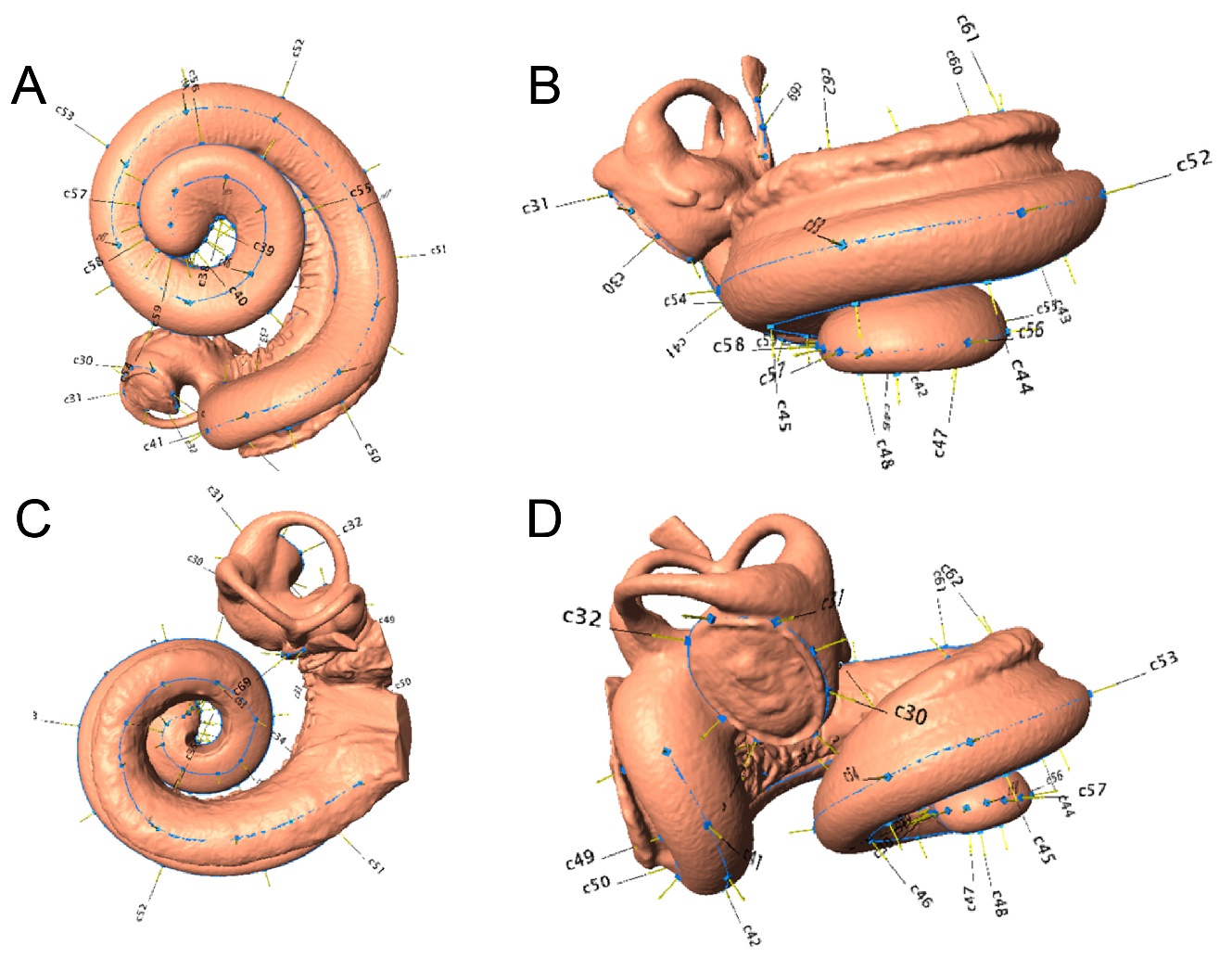


Figure S1. Cochlea of *Cephalorhynchus commersoniii* (NHMUK1952.6.20.4.2) in: (A) vestibular; (B) anterior; (C) dorsal; and (D) lateral views, showing placement of landmarks for this study.

**Chi-squared analyses results**

Table S2. Results of *χ*^2^ analysis. *Χ*^2^:  chi-squared value; df: degrees of freedom; bc: Bonferroni corrected *p*-value. Habitat1: all “riverine/nearshore” taxa are classed as “riverine” and “nearshore/oceanic” taxa are classed as “nearshore”; Habitat2: all “riverine/nearshore” taxa are classed as “nearshore” and “nearshore/oceanic” taxa are classed as “oceanic”; Feeding1: all “raptorial/suction” taxa are classed as “raptorial”; Feeding2: all “raptorial/suction” taxa are classed as “suction”. Significant ecological categories in bold.

|  | **Regime A** |  |  |  | **Regime B** |  |  |  | **Regime C** |  |  |  |
| --- | --- | --- | --- | --- | --- | --- | --- | --- | --- | --- | --- | --- |
|  | **x^2^** | **df** | ***p*-value** | **bc *p*-value** | **x^2^** | **df** | ***p*-value** | **bc *p*-value** | **x^2^** | **df** | ***p*-value** | **bc *p*-value** |
| Diet | 3.224 | 2 | 0.200 | 1 | 6.860 | 2 | 0.032 | 0.583 | 4.466 | 2 | 0.107 | 1 |
| Habitat 1 | 16.39 | 2 | 0.000 | **0.005** | 4.174 | 2 | 0.124 | 1 | 1.699 | 2 | 0.428 | 1 |
| Habitat 2 | 3.725 | 2 | 0.155 | 1 | 0.949 | 2 | 0.622 | 1 | 1.455 | 2 | 0.483 | 1 |
| Feeding 1 | 6.350 | 1 | 0.012 | 0.211 | 0.568 | 1 | 0.451 | 1 | 1.517 | 1 | 0.218 | 1 |
| Feeding 2 | 5.749 | 1 | 0.017 | 0.297 | 0.479 | 1 | 0.489 | 1 | 1.335 | 1 | 0.248 | 1 |
| Dive type | 8.908 | 2 | <0.001 | **<0.001** | 2.268 | 2 | 0.028 | 0.517 | 3.478 | 2 | 0.051 | 0.918 |

**3 PC & 4 PC Analyses Results**

*SURFACE analyses*

Table S3. Results of the SURFACE analysis using 3 PCs. Parameters were found by the evolutionary models fitted to the evolution of cochlear shape in toothed whales described by PC1, PC2 and PC3. Abbreviations: α, rate of adaptation to optima; *t*_1/2_,; σ^2^, rate of stochastic evolution; θ, optimum trait value for each regime.

| Parameter | Value |  |  |
| --- | --- | --- | --- |
| AICc | -547.6001 |  |  |
| Phenotypic regimes | 6 |  |  |
| Pheno reg shifts | 9 |  |  |
| Conv pheno reg | 3 |  |  |
| Conv reg shifts | 6 |  |  |
| Conv fraction | 0.66 |  |  |
|  | PC1 | PC2 | PC3 |
| α | 0.3055731 | 3.5860466 | 0.2034437 |
| *t*_1/2_ | 2.2683512 | 0.1932901 | 3.4070705 |
| σ^2^ | 0.000394199 | 0.00459924 | 0.000389555 |
| θ_a_ | 0.06475327 | 0.05540134 | 0.054914713 |
| θ_b_ | 0.05525293 | -0.0979297 | 0.424337912 |
| θ_c_ | 0.10949494 | 0.17117225 | 0.105221289 |
| θ_d_ | -0.12552789 | -0.0105983 | 0.016383934 |
| θ_f_ | 0.01960091 | -0.0127686 | -0.01536403 |
| θ_g_ | -0.07812261 | 0.13443546 | -0.00662119 |

Table S4. Results of the SURFACE analysis using 4 PCs. Parameters were found by the evolutionary models fitted to the evolution of cochlear shape in toothed whales described by PC1, PC2, PC3 and PC4. Abbreviations: α, rate of adaptation to optima; *t*_1/2_, expected time to evolve halfway to an optimum; σ^2^, rate of stochastic evolution; θ, optimum trait value for each regime.

| Parameter | Value |  |  |  |
| --- | --- | --- | --- | --- |
| AICc | -723.6117 |  |  |  |
| Phenotypic regimes | 7 |  |  |  |
| Pheno reg shifts | 9 |  |  |  |
| Conv pheno reg | 2 |  |  |  |
| Conv reg shifts | 4 |  |  |  |
| Conv fraction | 0.44 |  |  |  |
|  | PC1 | PC2 | PC3 | PC4 |
| α | 0.2126589 | 0.2834478 | 0.1456725 | 0.1111551 |
| *t*_1/2_ | 3.259431 | 2.445414 | 4.758257 | 6.235857 |
| σ^2^ | 0.00028452 | 0.00045942 | 0.00030189 | 0.000159964 |
| θ_a_ | -0.12532507 | -0.01065699 | 0.016172771 | -0.017275535 |
| θ_b_ | 0.06922345 | -0.17092699 | 0.556174253 | 0.630543914 |
| θ_c_ | 0.12299196 | 0.24896805 | 0.113935723 | -0.047773283 |
| θ_d_ | -0.07552558 | 0.14403766 | -0.008966596 | 0.069964052 |
| θ_e_ | 0.0868295 | -0.06262475 | -0.077356961 | -0.364601342 |
| θ_g_ | 0.06390308 | 0.0377028 | 0.054711786 | -0.045510447 |
| θ_h_ | 0.01874895 | -0.01094644 | -0.015387493 | 0.002121349 |

*C-metric analyses*

Table S5. C_1_ - C_4_ convergence measures and *p*-values using 3 PCs. *P*-values were derived from 1000 simulations to test the hypothesis that the observed values are greater than random simulations based on Brownian motion. Significant values in bold.

| Regime A | C1 | C2 | C3 | C4 |
| --- | --- | --- | --- | --- |
| C-value | 0.68877399 | 0.12592615 | 0.39373833 | 0.02373326 |
| P-value | **0** | **0** | **0** | **0** |
|  |  |  |  |  |
| Regime B | C1 | C2 | C3 | C4 |
| C-value | 0.849605 | 0.2225559 | 0.5308192 | 0.4826925 |
| P-value | **0** | **0** | **0** | **0** |
|  |  |  |  |  |
| Regime C | C1 | C2 | C3 | C4 |
| C-value | 0.256274777 | 0.031864892 | 0.056861626 | 0.006005566 |
| P-value | 0.07792208 | **0.04195804** | 0.31368631 | 0.30669331 |

Table S6. C_1_ - C_4_ convergence measures and *p*-values using 4 PCs. *P*-values were derived from 1000 simulations to test the hypothesis that the observed values are greater than random simulations based on Brownian motion. Significant values in bold.

| Regime D | C1 | C2 | C3 | C4 |
| --- | --- | --- | --- | --- |
| C-value | N/A | N/A | N/A | N/A |
| P-value | N/A | N/A | N/A | N/A |
|  |  |  |  |  |
| Regime E | C1 | C2 | C3 | C4 |
| C-value | 0.7049156 | 0.2275502 | 0.4730401 | 0.4382331 |
| P-value | **0.000999001** | **0.000000000** | **0.000000000** | **0.000000000** |

*Wheatsheaf analyses*

Table S7. Results of the Wheatsheaf index analysis using 3 PCs. WI: Wheatsheaf index.

| Conv reg | WI value | P-value | 95% CI |
| --- | --- | --- | --- |
| Regime A | 1.022464 | 0.747 | 0.9526044 - 1.089544 |
| Regime B | 2.194074 | 0.34 | 2.092535 - ∞ |
| Regime C | 1.472754 | 0.679 | 1.404596 - 2.749955 |

Table S8. Results of the Wheatsheaf index analysis using 4 PCs. WI: Wheatsheaf index.

| Conv reg | WI value | P-value | 95% CI |
| --- | --- | --- | --- |
| Regime D | 2.420819 | 0.292 | 2.324896 - ∞ |
| Regime E | 0.951743 | 0.14 | 0.9319229 - 0.9725553 |


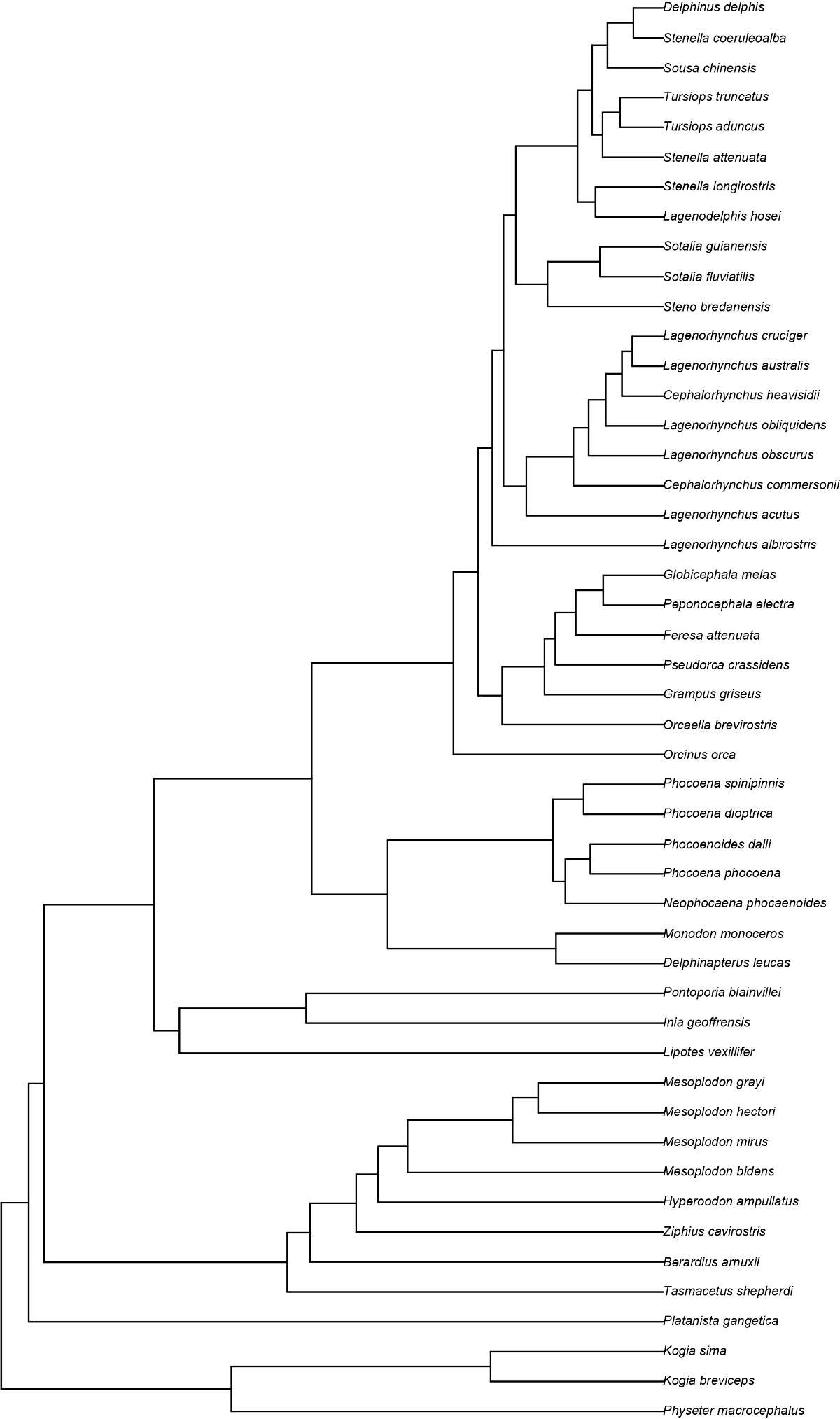


Figure S2. Cladogram showing the phylogenetic relationships of the taxa in this study.
